# An international patient survey of RYR1-related diseases: The patient perspective on clinical trial participation

**DOI:** 10.1177/22143602261493651

**Published:** 2026-09-29

**Authors:** Lizan Stinissen, Sanne A.J.H. van de Camp, Brentney Simon, Lindsay Goldberg, Baziel G.M. van Engelen, Kirsten E. Bevelander, Wija Oortwijn, Nicol C. Voermans, on behalf of RYR1-RD patient survey study group

**Affiliations:** 1 Department of Neurology, Donders Institute for Brain, Cognition and Behavior, Radboud University Medical Center, Nijmegen, The Netherlands; 2 503226 The RYR-1 Foundation, Pittsburgh, PA, USA; 3 Research Institute for Medical Innovation, Primary and Community Care, Radboud University Medical Center, Nijmegen, The Netherlands; 4 Research Institute for Medical Innovation, Science department IQ Health, Radboud University Medical Center, Nijmegen, The Netherlands

**Keywords:** *RYR1*-related diseases, patient perspective, patient preferences, trial experiences

## Abstract

**Background and objective:**

*RYR1*-related diseases (*RYR1*-RD) are a group of genetic conditions characterized by early-onset muscle weakness, causing functional impairments. An increasing number of clinical trials is on the horizon. This study aims to improve trial readiness by characterizing the patient perspective on clinical trial participation.

**Methods:**

In collaboration with *RYR1*-RD patients and patient representatives we adapted an existing facioscapulohumeral muscular dystrophy (FSHD) survey and conducted the resulting international patient survey in 2025. We used descriptive statistics to analyze the patient characteristics and personal experiences and expectations in clinical trials.

**Results:**

In total 146 participants (of whom 30 with previous trial experiences), residing mainly in the Netherlands or the United States, completed the survey. Symptoms that caused the greatest difficulties in daily life, including muscle weakness, fatigue, and impaired mobility, were seen as important outcomes for future trials. The possibility to receive the trial drug and results when published were reported as facilitators of trial participation. There was a strong demand for receiving updates on ongoing and upcoming trials. Fear of side effects and travel time were reported as barriers.

**Conclusion:**

A clear understanding of patients’ expectations and preferences is important for relevant future clinical trials to better align with patients’ needs.

## Introduction

*RYR1*-related diseases (*RYR1*-RD) are a group of genetic conditions characterized by early-onset muscle weakness, leading to functional impairments. The *RYR1* gene encodes the primary calcium release channel of the sarcoplasmic reticulum. Pathogenic variants in the *RYR1* gene cause a broad range of congenital myopathies (CMYOs) and can also be associated with malignant hyperthermia susceptibility (MHS), with a wide associated clinical spectrum.^1,2^

There are no curative treatments for *RYR1-*RD, however, there are several preclinical and natural history studies ongoing and clinical studies likely upcoming.^1–3^ For example, there have been two clinical trials.^3,4^ A phase two, randomized controlled trial of N-acetylcysteine (NAC) demonstrated that NAC was safe and well-tolerated in *RYR1*-RD patients. However, NAC did not reduce oxidative stress or significantly improve physical endurance.^3^ A phase one, open-label, dose-escalation trial with S48168 (ARM210) demonstrated favorable safety, tolerability, pharmacokinetics, and target tissue penetration in *RYR1-*RD affected individuals. Most trial participants reported decreased fatigue and increased proximal muscle strength, but these findings warrant further investigation in a randomized, masked, placebo-controlled trial.^4^

Future clinical trials call for optimal trial readiness of all relevant stakeholders involved. This is especially important in rare myopathies, due to smaller participant populations, diagnostic delays, clinical overlap with other myopathies, unidentified genetic causes and limited natural history data.^5^

Research on trial preferences in another neuromuscular disease, facioscapulohumeral muscular dystrophy (FSHD), highlighted the importance of patient preference studies while also noting the lack of research on this topic for neuromuscular diseases in general.^6^

In a previous study, we examined the perspectives of 227 patients and caregivers on living with *RYR1*-RD, including the diagnosis, impact of the disease, physical activity, treatment, and participation in clinical studies. This patient-initiated study recommended that the expectations, barriers, and facilitators of trial participation should be studied in more detail.^7,8^

Therefore, the aim of this follow-up study was to provide insight into patients’ perspectives on participation in future clinical trials, including the symptoms they considered most burdensome, facilitators and barriers to participation, and their willingness to engage in specific trial components. This is expected to contribute to better aligning future trials with patients’ needs.

## Methods

### Study design

We conducted an international survey for patients diagnosed with an *RYR1*-RD. The online survey was available from March 10^th^ until May 5^th^ 2025. This survey was created in close collaboration with The RYR-1 Foundation, and members of the study group, consisting of clinical researchers, patients, family members, and patient representatives. The survey was based on a previous questionnaire performed by FSHD Europe, which gave permission for its use and adjustment.^6^ Questions were modified to better align with *RYR1-*RD patients. The adjusted survey was discussed within the study group, and we asked for reflections from a board member of the pharma company ARMGO, now known as RyCarma, who provided minor comments. The survey was piloted by two patients and they indicated that no changes were needed. The final survey consisted of 58 questions regarding clinical information about the current condition and management of *RYR1*-RD, thoughts and experiences of clinical trials, and expectations for future trials. The full survey is presented in S9. The survey was made available in six languages; English, Dutch, Spanish, Portuguese, French, and German. These were based on the FSHD questionnaire,^6^ and Portuguese was added to reach patients in South America. For the translations we used ChatGPT version 4o. The initial translations were reviewed and corrected where needed by native-speaking patients. It was possible to save the survey partway through and proceed at a later time.

### Inclusion criteria

The inclusion criteria included having received a medical diagnosis of an *RYR1*-RD by a healthcare professional, and being able to understand one of the available languages; English, Spanish, Portuguese, German, French, or Dutch. For individuals ≤16, parents or legal guardians were invited to complete the survey together with their child.

### Recruitment

Open convenience sampling was used by having the survey open to everyone with a medical diagnosis of an *RYR1-*RD and to their caregivers. The invitation to take part in the survey was shared multiple times through the networks of the study group, the newsletters and social media of several patient organizations including The RYR-1 Foundation, ZNM-ZusammenStark! e.V., Spierziekten Nederland, and during The RYR-1 Foundation webinar on the patient perspective and participation.^9^ The invitation was also shared with patients that were known at the Radboudumc and the Amalia Children’s Hospital in the Netherlands. Additionally, the survey was distributed through the network of the Patient and Citizen Interest Group of Health Technology Assessment International, where it was shared with the Taiwan Foundation for Rare Disorders. Participation was voluntary and informed consent for participation was asked at the start of the survey. The survey was distributed through CastorEDC. If patients had signed up for the survey, but had not yet started or finished it, reminders were sent to encourage participants to complete the survey. Incomplete surveys were not included in the data analysis.

### Ethical considerations

A waiver was provided by the Medical Ethical Review Committee East-Netherlands and Committee Human Research of Radboud university medical center (Radboudumc) (File number 2024-17830). The study was performed in accordance with the good clinical practice guidelines of the Radboudumc.

### Collected data

In the survey, questions were asked regarding the following topics; demographic information, information on current condition and management of *RYR1*-RD, previous experience with clinical trials, encouraging and discouraging factors in clinical trials, logistical aspects of clinical trials, expectations of future clinical trials, and concerns for the future.

### Statistical analysis

The data analysis was performed using SPSS (SPSS version 29, IBM, Armonk, New York). Graphs were prepared in Graphpad Prism software version 10 (Graphpad Software, San Diego, CA, USA). Median scores [interquartile range] were used to analyze the patient characteristics and personal experiences and expectations in clinical trials. The percentages of patients reporting certain aspects are depicted in the graphs. Trials often include only children or only adults, and only ambulatory patients. Therefore, we divided the participants into two age groups, <18 years old and ≥18 years old. The results for the patients <18 years old are answers provided by their parents. The groups were also stratified based on their ambulatory status (normal, I can walk independently with aids and do not use a wheelchair, I can walk independently with aids and I use a wheelchair outdoors, I use the wheelchair indoors and outdoors, but can walk short distances, or wheelchair dependent). In addition, the group was divided based on whether they would consider participating in future clinical trials. These groups were compared to assess if symptoms, and facilitators and barriers were different for those groups. The group differences were assessed using Chi Squared tests. The results were interpreted within the study group to discuss the most important and relevant outcomes.

## Results

### Participant information

The survey was completed by 152 participants. Six respondents reported not having a diagnosis provided by a healthcare professional and were therefore excluded, resulting in a total of 146 participants, including 98 females and 48 males (Table 1). The majority of the participants were patients (78%), some were a caregiver (16%) or both a patient and caregiver (6%). The survey respondents originated from 19 countries (Table S1). The countries that were most represented included the United States (30%), the Netherlands (22%), Germany (12%) and Mexico (7%). The ages of the participants ranged from 0 to 83 years, the majority were between 50 and 59 years old (21%).

**Table 1.** Information about current clinical condition.

Number of participants	146
Patient or caregiver (n)	114 P (78%)
	24 C (16%)
	8 both (6%)
Current age (median years [IQR])	47 [28-59]
Sex (n)	98 F (67%)
	48 M (33%)
Ambulatory status (n (%))	
Normal	56 (38%)
Being able to walk independently with aids, not using a wheelchair	41 (28%)
Being able to walk independently with aids, using a wheelchair outdoors	22 (15%)
Using a wheelchair indoors and outdoors, walking short distances (with or without aids)	7 (5%)
Wheelchair dependent	20 (14%)

IQR: interquartile range, P: patient, C: caregiver.

Most participants were diagnosed between the ages of 0 and 4 years old (25%) (Figure 1). The severity was scored on an ordinal scale with 45 participants mildly affected (31%), 29 moderately affected (20%), and 3 individuals severely affected (2%). Out of the different age groups, participants aged between 0 and 4 years old were the most severely affected individuals (57%) (Figure 2A). Over time, a majority of participants responded that the disease had gotten minimally worse over 6 months (43%), or had not changed (35%) (Figure 2B).

**Figure 1.**
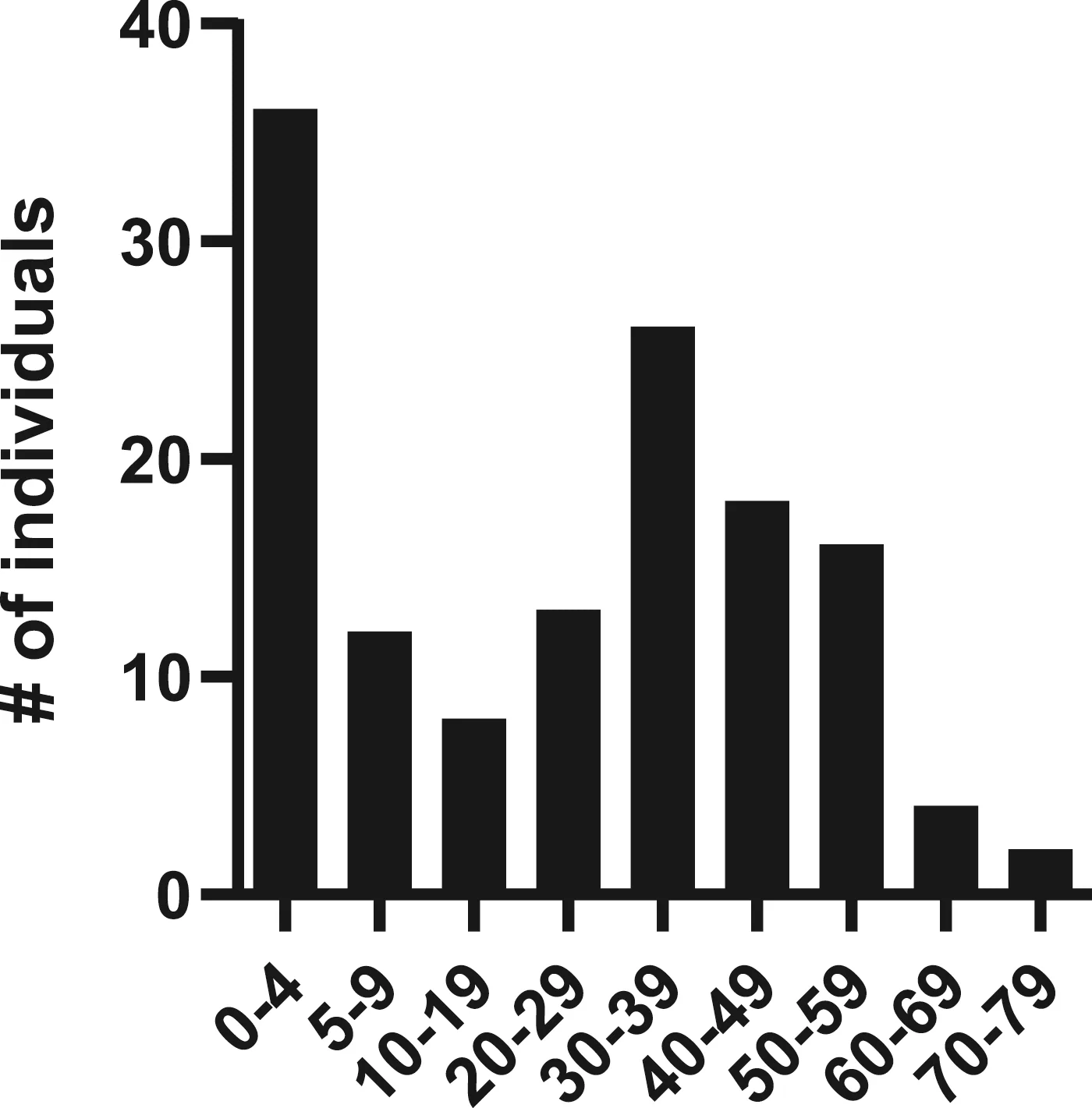
Age at diagnosis. The x-axis depicts the different age groups. The y-axis depicts the number of participants for each age group.

**Figure 2.**
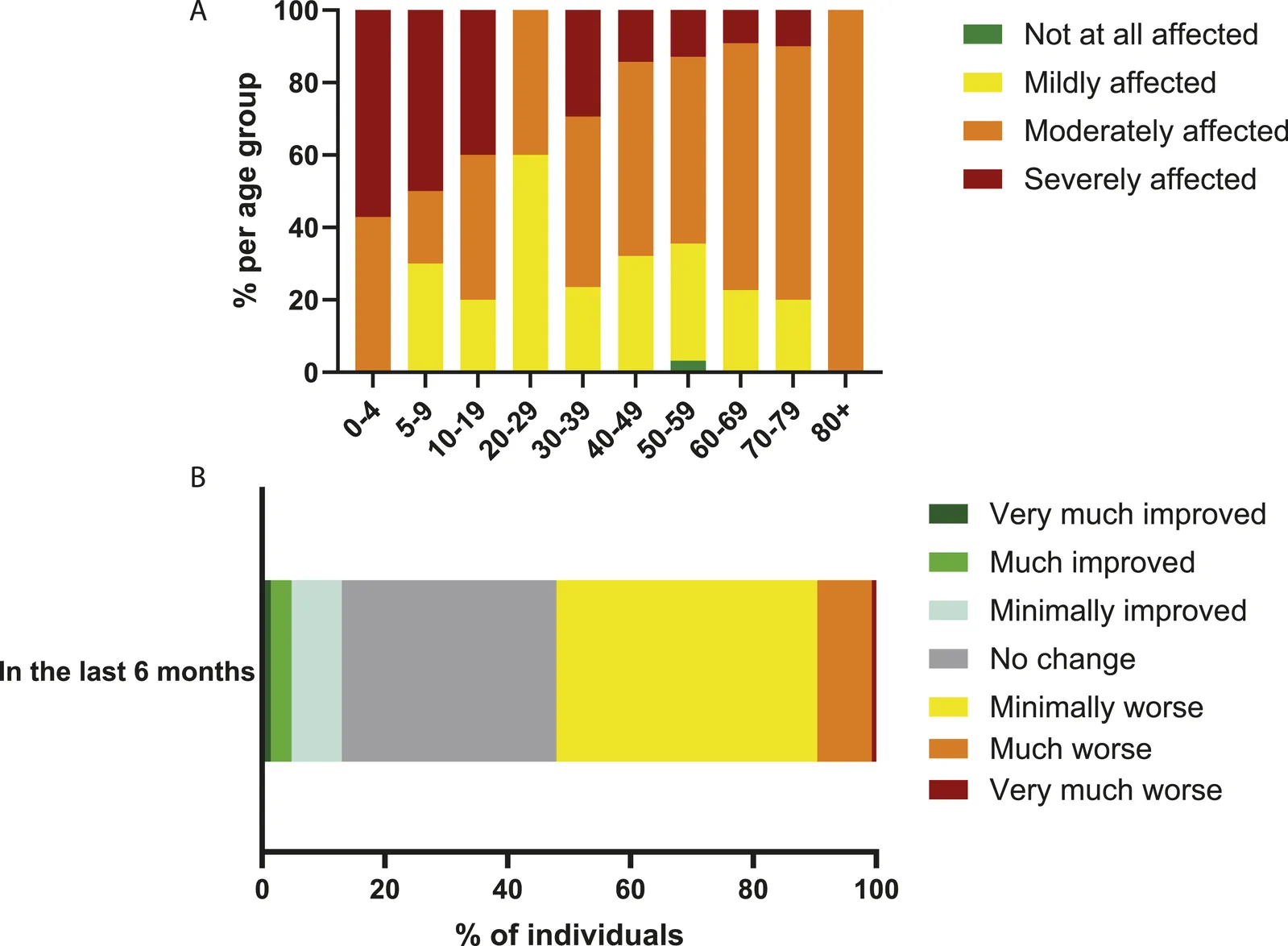
Reported severity at current age (A) and change in severity over time (B) (A): For each age group it is depicted what percentage of the participants are not at all affected (green), mildly affected (yellow), moderately affected (orange) or severely affected (red). (B): The reported change in severity over a period of 6 months. Darker green indicates less affected/greater improvement in severity, and darker red indicating severely affected/greater increase in severity.

Participants reported general muscle weakness as the symptom associated with the greatest difficulties in daily life. This was followed by fatigue, energy and endurance, and impaired mobility (Figure 3). Individuals whose walking ability was unaffected reported the fewest overall difficulties in daily life. Participants identified ‘lack of energy to work or live as desired’ as their primary future concern, followed by ‘loss of independence’ and ‘loss of mobility’ (Figure 4).

**Figure 3.**
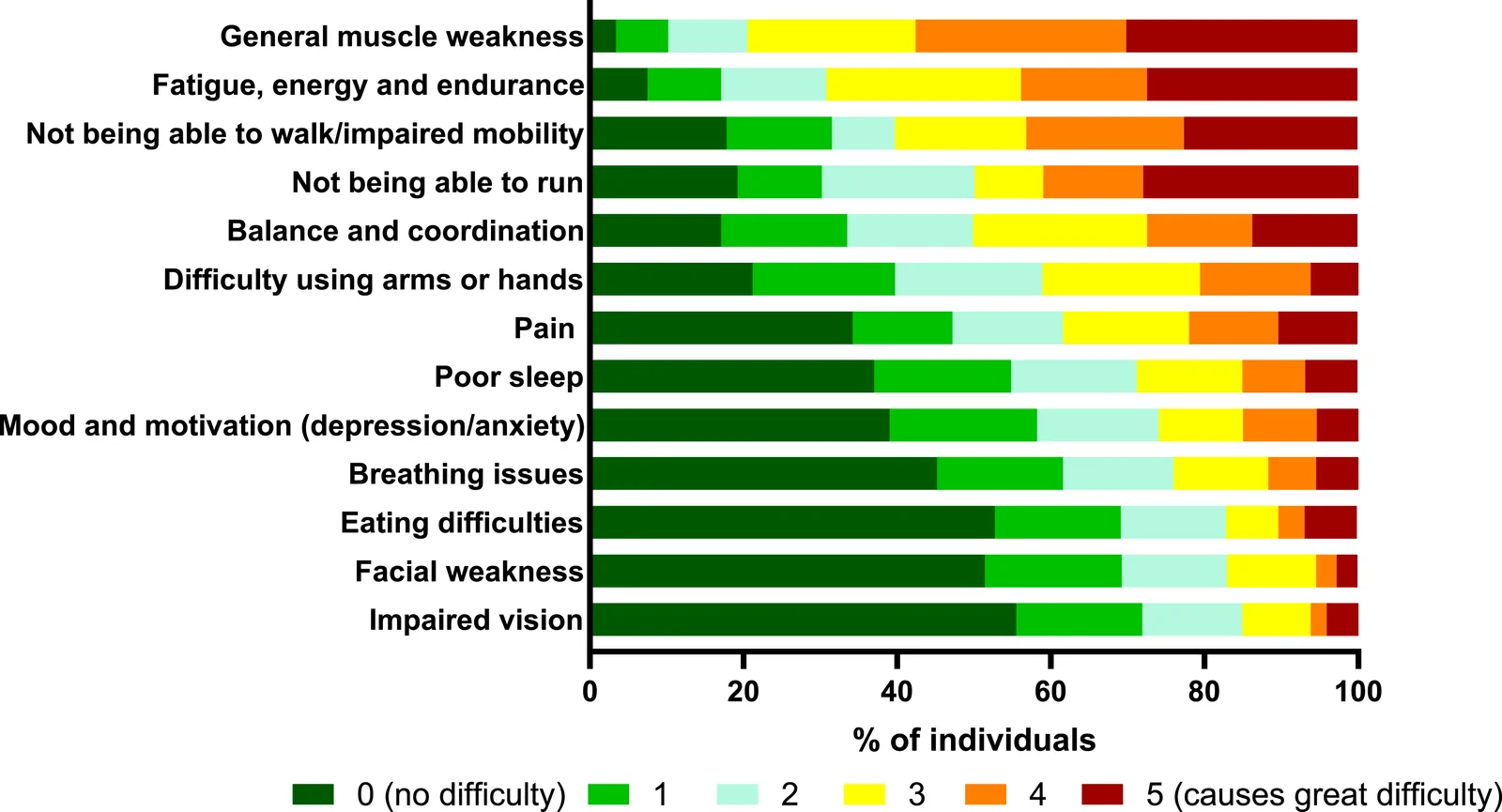
Symptoms causing difficulties in daily life. Participants rated each symptom from 0 (dark green; no difficulty) to 5 (red; causes great difficulty). The symptoms are ranked from highest median score (top) to lowest median score (bottom).

**Figure 4.**
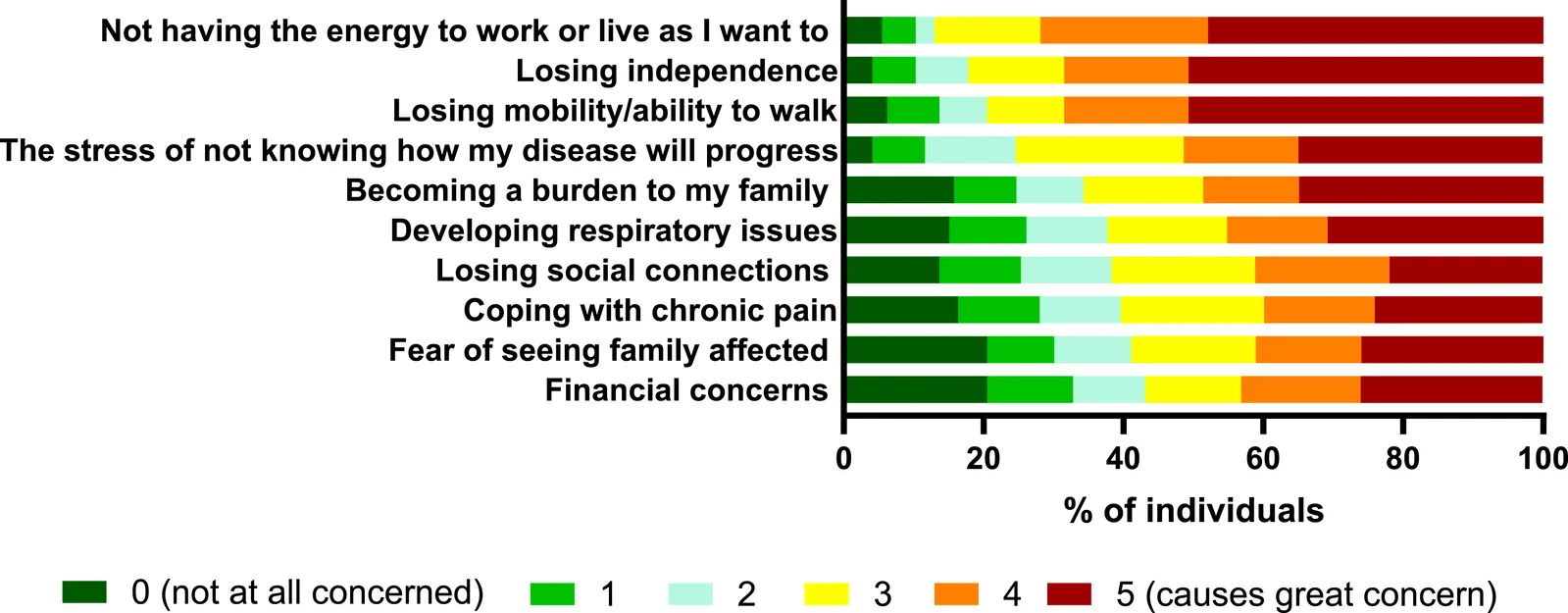
Concerns for the future. Participants rated each concern from 0 (dark green; not at all concerned) to 5 (red; causes great concern). The concerns are ranked from highest median score (top) to lowest median score (bottom).

Most patients (92%) were using some form of support measure (either medicinal, or non-medicinal) (Table S1). However, only a small portion of patients stated that their medications or support measures work to a great extent (11%) (Figure S2).

### Thoughts and experiences of clinical trials

30 individuals (21%) had participated in a clinical trial before. Among these, 18 individuals (60%) had a positive experience throughout this trial, 8 participants (27%) had a neutral experience, and 4 (13%) had a negative experience. When participants were asked if they would consider participating in a clinical trial, the majority reported ‘yes’ (69%), about one third reported ‘not sure’ (28%), and a few reported ‘no’ (3%).

Looking into encouraging factors for trial participation, the most important facilitators included 1) access to new investigational product/therapy, 2) access to trial results when published, 3) benefits for the *RYR1*-RD community (Figure 5A). In turn, the most discouraging factors (barriers) for trial participation were 1) fear of side effects from treatment, 2) facility of the clinical trial being far away, 3) lack of flexibility in appointment times (Figure 5B).

**Figure 5.**
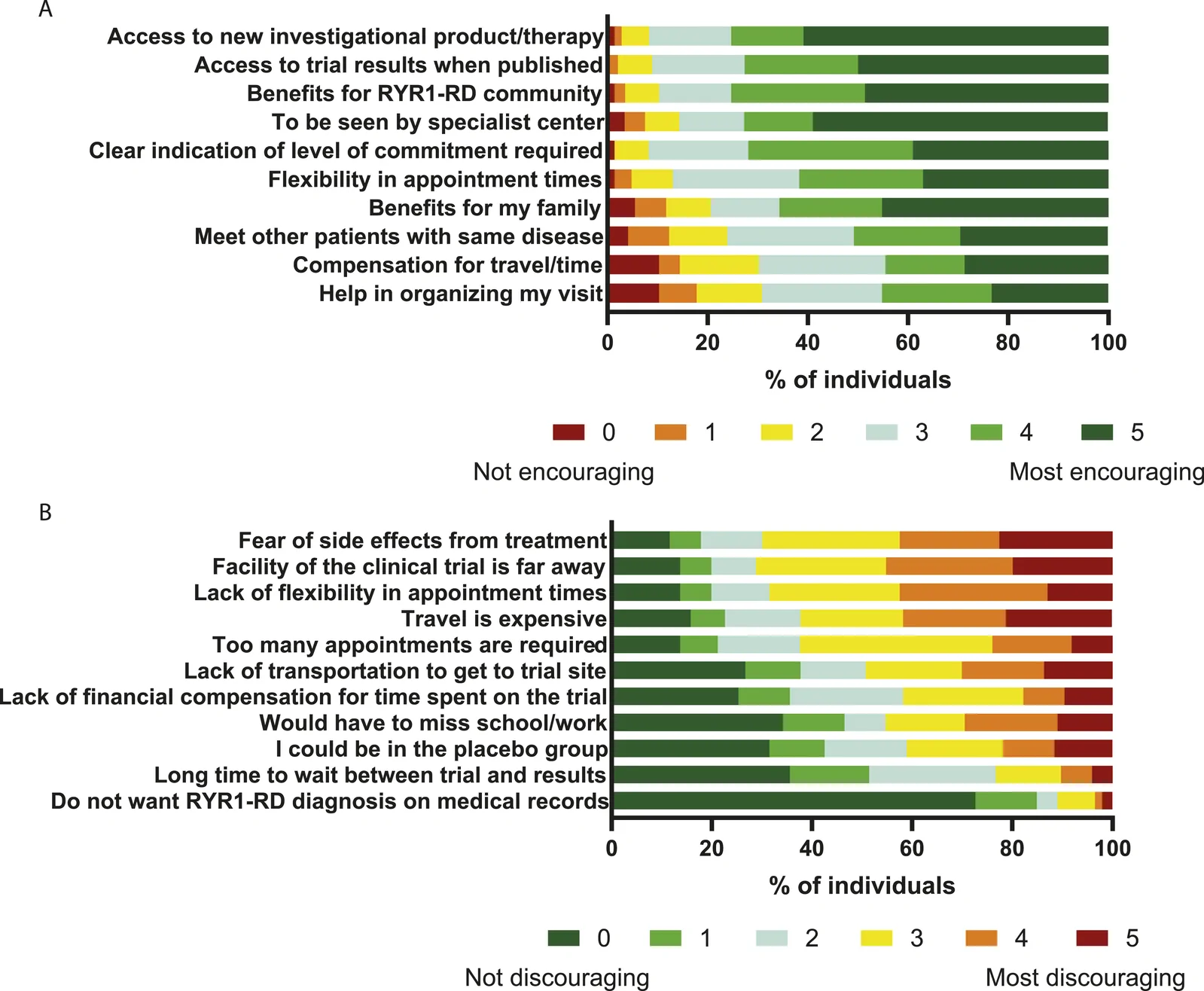
Encouraging and discouraging factors for participating in a clinical trial. (A): Encouraging factors. Participants reported each factor from 0 (red; not encouraging) to 5 (dark green; most encouraging). The factors are ranked from highest median score (top; most encouraging) to lowest median score (bottom; least encouraging). (B): Discouraging factors. Participants reported each factor from 0 (dark green; not discouraging) to 5 (red, most discouraging). The factors are ranked from highest median score (top; most discouraging) to lowest median score (bottom; least discouraging).

### Differences between groups for facilitators and barriers

After stratification by willingness to participate in future clinical trials, participants who would consider trial participation reported more positive attitudes towards facilitators, while those who were unsure or unwilling expressed more negative attitudes towards barriers.

One facilitator and one barrier seem to have scored differently between ambulatory statuses (p<0.05). Less ambulant groups have a higher score for the facilitator ‘help in organizing my visit’. Participants with normal ambulation and participants who can walk independently with aids and use a wheelchair outdoors report fear of side effects less often as a barrier.

When stratifying for age, fear of side effects was more discouraging for pediatric patients (median score of 4.00 vs 3.00) and meeting other patients was more encouraging for pediatric patients (median score of 5.00 vs 3.00). These answers were indicated by the parents or caregivers of the pediatric patients.

### Receiving information

When asked how participants received information about *RYR1-*RD related topics and updates on clinical trials, most individuals responded that they received this through The RYR-1 Foundation (53%), through their national patient organization (16%), or via their treating physician (13%). Some participants (14%) did not receive any information. The majority of these individuals found the information they received sufficient. However, for 19% of the participants this information was insufficient. There was a strong demand for information from the physician, more regular updates regarding trials and research developments, and being able to connect with individuals in similar situations.

### Expectations for future research

Participants were instructed to select important outcomes in the survey. The most often selected important outcomes of a new therapy for participants were regaining strength (62%), stopping the progression of the disease (51%), reduction in fatigue (50%), and improved mobility (40%) (Figure 6). Additionally, participants were asked to select symptoms that they would like to see improved first. The symptoms that patients most often selected were fatigue (62%) and general muscle weakness (62%) (Figure S5). In addition, the most important social benefits selected by participants were increased independence (65%), and participation in sports and hobbies (58%) (Figure S6).

**Figure 6.**
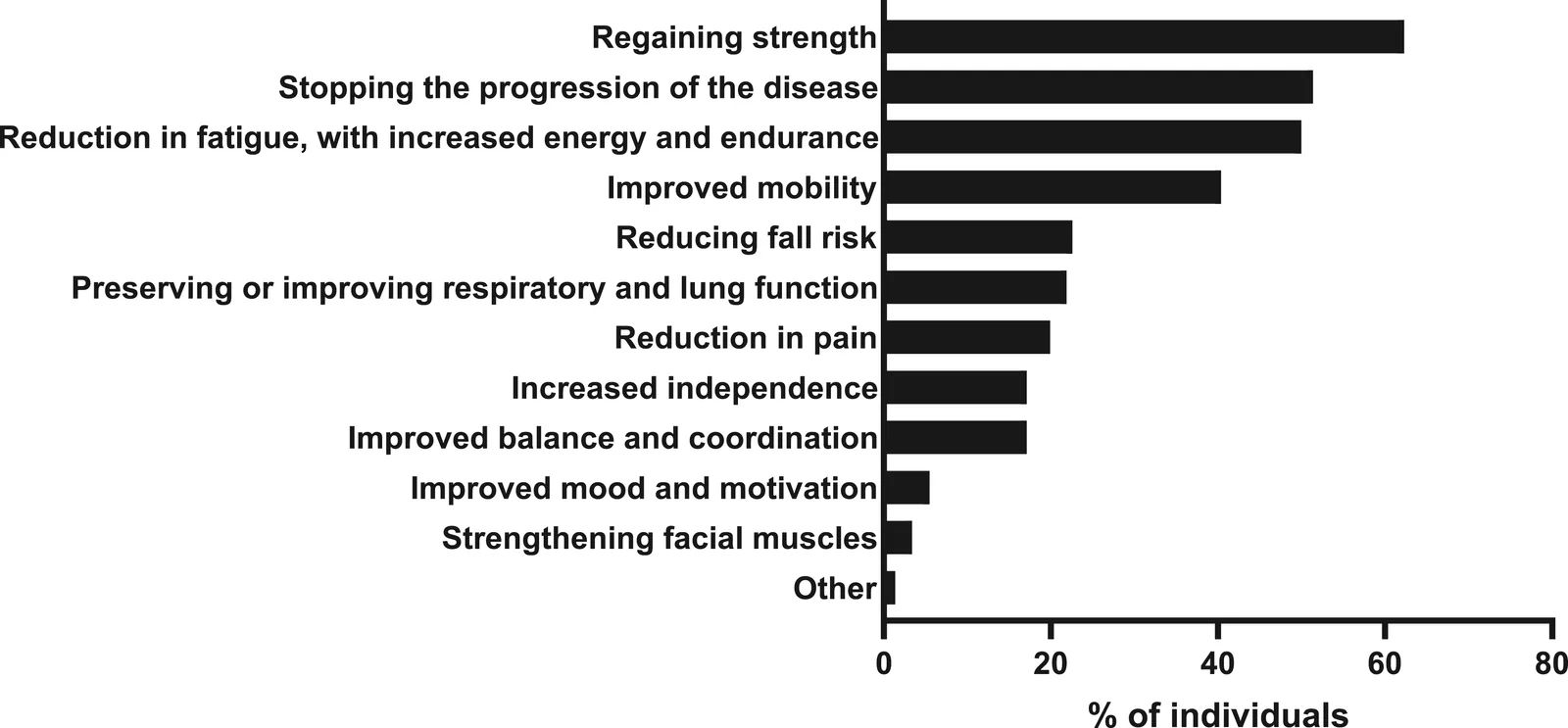
Most important outcome of a new therapy. The x-axis depicts the percentage of participants that chose that outcome.

Participants were most worried about the side effects liver/kidney problems (50%), followed by behavioral side effects (40%), heart-related effects (39%), and stomach/gut effects (38%) (Figure S7).

Possible examinations that participants would be willing to undergo or participate in as part of a trial are depicted in Figure S8. The participants indicated willingness to undergo most examinations, except for needle or open muscle biopsy. Other practical aspects of trials that patients were asked about can be found in Figures S3 through S9. Figure S3 illustrates the tolerated MRI duration, up to an hour was the most reported answer (39%). Figure S4 demonstrates the recovery time from traveling to the trial site, not traveling on the same day was reported by most participants (51%). Other practical aspects included travel time (Figure S9A), in which the majority (51%) indicated a willingness to travel five or more hours. Almost all participants (93%) would be willing to stay overnight (Figure S9B), and almost half of the participants (48%) would be willing to be four or more nights away from home (Figure S9C). More than half of the participants (56%) would require a companion to travel with them (Figure S9D).

Finally, several topics for future research were proposed, with the most frequently reported being stopping the progression, improving strength, and finding a cure. Other topics that were mentioned involved diet, improving mobility, exercise, fatigue, and sleep. In addition, research on respiratory function and pain was noted. Moreover, topics on quality of life, mental aspects, coping, support and impact on carers and patients’ livelihoods were also described as important future topics.

## Discussion

This survey captured participants’ perspectives on clinical trials, including the symptoms they found most burdensome, the facilitators and barriers to trial participation, and the specific trial components they would be willing to engage in. This study is a follow-up to our first study in which we examined the impact of living with an *RYR1*-RD from the patient and caregiver perspective.^8^ The previous study was patient-initiated and also in the current study we designed and disseminated the survey, and interpreted its results in collaboration with patients and patient representatives. This allowed us to include questions on topics prioritized by patients. Research has shown that taking the patients’ perspective into account in clinical trial design enhances recruitment and patient-relevant outcomes.^10–12^

Our main findings show that participants feel that the greatest difficulties in daily life are caused by general muscle weakness, fatigue and impaired mobility. These symptoms are in line with participants’ concerns about the future and what symptoms participants would like to see improved first. A previous survey also revealed *RYR1*-RD to be associated with severe fatigue.^13^ Studies in other NMDs have shown similar results.^6,14,15^ These symptoms should therefore be taken into consideration in future trial design. For example, future protocols for study visits should be limited, to decrease the influence of fatigue. Ensuring alignment with what is achievable for patients is important. In addition, a measurement on experienced fatigue, for example the CIS fatigue, could be incorporated in future clinical trials.

Almost all participants reported using one or multiple medications and/or additional support measures. However, the minority of patients indicated that these support measures control their symptoms to a great extent. This highlights the importance of conducting clinical trials to develop and investigate new therapies, as well as other studies to enhance the effectiveness of other support measures.

Several aspects should be considered when designing study protocols, as identified by participants as important for future clinical trials. The majority of the participants are willing to participate in a clinical trial. Important aspects that would encourage participation were access to the investigational product/therapy, access to trial results when published, and being seen by a specialist center. This aligns with findings in other studies on experiences in trial participation in centronuclear myopathy (CNM) and FSHD patients.^16,17^ In these studies participants had realistic expectations about the drug efficacy in a clinical trial, with hope for improvement in strength and an overall better quality of life. Such expectation management is crucial, as it requires balancing between possible therapy effects and risks. Open communication before, during, and after the trial, was deemed important. This included communication from the trial sites about participants’ expectations and trial visits, as well as updates from the sponsor about the trial progress. When designing a trial, it is essential to organize trial visits according to the patients’ needs.^16,17^

The respondents also reported important barriers to trial participation, including fear of side effects from the treatment, facility of the clinic being far away, and lack of flexibility in appointment times. Even though the facility of the clinic being far away is stated as a barrier, most participants are still willing to travel more than 5 hours. These findings on barriers are consistent with those obtained using the FSHD questionnaire, which served as the basis for this study.^6^ In addition, this is in line with our previous study on living with *RYR1-*RD, in which impact on daily life and safety concerns were reported as the main barriers to trial participation.^8^ While *RYR1-*RD and FSHD are both inherited myopathies, they differ in onset and clinical presentation. In FSHD, scapular and facial weakness is predominantly involved.^18^ The disease typically manifests during adolescence and early adulthood. *RYR1*-RD is a type of CMYO, characterized by early onset proximal weakness with significant respiratory involvement.^2^ Despite the differences in presentation, they show similarities in patient trial experiences and expectations. Multicenter clinical trials could increase accessibility by reducing the travel distance to a study site. Although most participants indicated that they would be willing to travel for several hours, including study sites in different regions may enable participation among patients for whom longer travel distances remain a barrier.

When designing a trial it is important to take note of the patients to be included. Arranging transportation for patients, especially for the patients who are less ambulant, should be incorporated when designing a trial. When dividing between pediatric and adult participants, meeting other patients with the same disease was more encouraging for pediatric participants than for adult participants. Even though meeting other patients is often not allowed in trials, we included this as an encouraging factor because patients often reported interest in this. Precisely for this reason it is important that patients have an opportunity to get in touch with other patients outside of the trial. Patient organizations can have a vital role in this.

Patient organizations can play an important role in clinical research by providing insight into the experiences, needs, and burdens of patients and their families. Incorporating these perspectives into the design of future clinical trials may help reduce the burden of participation, support appropriate planning of study visits, and improve patient recruitment and retention.^16^ We consider the collaboration with patients and patient organizations and the dissemination of the survey across various platforms strengths of this study. Through translation in different languages and wide distribution of the survey, we aimed to maximize outreach. However, most participants originated from the Netherlands and the United States, therefore, the results might not be generalizable for other countries. In the future, wider dissemination of the survey through further international collaboration could be beneficial, which also allows for a comparison between countries. In addition, recruitment through patient organizations, social media, and webinars may have resulted in a sample that was more engaged with the RYR1-RD community and more aware of clinical research than the broader RYR1-RD population. Furthermore, we do not know how many patients have seen the invitation to participate and decided not to participate. Therefore it is unclear to what extent the responses are representative of the broader patient population. Consequently, willingness to participate in clinical trials and interest in trial-related information may have been overestimated. Another limitation was the interpretation bias, since the survey was self-reported. For example, for a few questions the participants were asked to provide up to three answer options. Nevertheless, in some cases, participants selected more than three answers. Interpretation of the questions regarding ‘access to trial results’ could have had multiple interpretations such as access to published aggregate data or individual-level results. Returning individual data is not standard of practice, but is valued by patients,^19^ and publishing aggregate data often takes 2 years after trial completion.^20^ It is important to be aware that patients find it important to receive results, either individual or group level, when analyzing the results. In our previous study among patients with FSHD, participants particularly valued receiving updates on their individual disease progression and on the overall progress and planning of the trial.^17^ Additionally, the survey duration was approximately 45 minutes. We noticed some participants starting the survey, but not finishing it. The length of the survey could be one of the causes even though the survey was piloted. Finally, the survey was also open to children. The instructions were for parents to fill out the survey on behalf of their child. It is possible that the parents’ perspectives are different compared to the child’s experiences. More broadly, responses from patients, caregivers, and respondents identifying as both were generally analyzed as a pooled group. As the caregiver subgroups were too small to allow reliable stratified analyses, potential differences between patient and caregiver perspectives, including differences in perceived barriers, facilitators, and trial-related preferences, could not be fully explored.

## Conclusion

A clear understanding of patients’ expectations and preferences is essential for the design of future clinical trials. Our study identified several key aspects. Participants emphasized muscle weakness, fatigue and impaired mobility as the most important outcomes for future trials. The main facilitators of trial participation included possible access to the trial drug and results when published. There was a strong demand for receiving updates on ongoing and upcoming trials. The fear of side effects and travel time were reported as the main barriers to trial participation. Overall, participants indicated a desire for more frequent updates regarding trials and research developments. By prioritizing aspects that patients consider most critical, future trials can better align with patients’ needs, which is likely to enhance participant retention and improve data quality. This patient-centered approach enhances the relevance and effectiveness of potential treatments, being meaningful to those affected.

## Supplemental material

Supplemental material - An international patient survey of RYR1-related diseases: The patient perspective on clinical trial participation Supplemental material for An international patient survey of RYR1-related diseases: The patient perspective on clinical trial participation by Lizan Stinissen, Sanne A.J.H. van de Camp, Brentney Simon, Lindsay Goldberg, Baziel G.M. van Engelen, Kirsten E. Bevelander, Wija Oortwijn, Nicol C. Voermans in Journal of Neuromuscular Diseases

## Data Availability

Due to the nature of this research, participants of this study did not agree for their data to be shared publicly, so supporting data is not available. Researchers who are interested in the data set are welcome to approach the corresponding author.*
