# Supplementary material for: An international patient survey of RYR1-related diseases: The patient perspective on clinical trial participation
Source: J Neuromuscul Dis. 2026 Sep 29:22143602261493651. Online ahead of print. doi: 10.1177/22143602261493651 (PMC13624179; doi:10.1177/22143602261493651)
Supplement: Supplementary file 1 [file sj-pdf-1-jnd-10.1177_22143602261493651.pdf]

Supplemental data

Table S1 : Participant information

	N
Age at first symptoms (median years [IQR])	0 [0-4.5]
Age at diagnosis (median years [IQR])	27 [3.5-43.5]
Severity (n (%))	
Not at all affected	1 (1%)
Mildly affected	39 (27%)
Moderately affected	75 (51%)
Severely affected	31 (21%)
(Histopathological) phenotype (n (%))	
CCD	82 (56%)
MmD	10 (7%)
CNM	9 (6%)
MHS	8 (6%)
CFTD	4 (3%)
Other	13 (9%)
Unknown	20 (14%)
Inheritance (n (%))	
Autosomal dominant	49 (34%)
Autosomal recessive	42 (29%)
De novo/spontaneous	16 (11)
Unknown	39 (27%)
Origin (n (%))	
North America	48 (33%)
Latin America	19 (13%)
Western Europe	68 (47%)
Eastern Europe	1 (1%)
Middle East and Africa	2 (1%)
Asia and Oceania	8 (6%)
Highest educational level (n (%))	
Preschool	11 (8%)
Primary school	13 (9%)
Secondary school	16 (11%)
Vocational education and training	23 (16%)
College/university (Bachelor)	50 (34%)
College/university (Master)	26 (18%)
PhD/MD/JD (doctoral level)	7 (5%)
Employment status (n (%))	
(Volunteer)work fulltime (>32h/week)	56 (38%)
(Volunteer)work parttime (<32h/week)	20 (14%)
Retired	22 (15%)
Unable to work	21 (14%)
Still in school	27 (19%)
Respiratory status (n (%))	
Not affected	69 (47%)
Mildly affected	45 (31%)
Moderately affected (require non-invasive ventilation)	29 (20%)
Severely affected (require invasive ventilation)	3 (2%)
Medication	
Salbutamol/pyridostigmine	24 (16%)
Anti-anxiety medication	13 (9%)
Pain killers	25 (17%)

Other prescription medication	26 (18%)
Over the counter medications (paracetamol, ibuprofen, Tylenol, etc.)	40 (27%)
Dietary and herbal supplements	35 (24%)
Other	15 (10%)
Nothing	54 (37%)
Support measures	
Exercise	82 (56%)
Physical or occupational therapy	71 (49%)
Braces, splints, kinesio tape, etc.	29 (20%)
Mobility aids (such as a walker or wheelchair)	64 (44%)
Electric bike	30 (21%)
(Nocturnal) mechanical ventilation	18 (12%)
Surgery such as scoliosis surgery	16 (11%)
Diet modification	25 (17%)
Counselling/therapy	23 (16%)
Adjustments to the house	43 (30%)
Adjustments to the car	20 (14%)
Other	14 (10%)
Nothing	15 (10%)

IQR: interquartile range, CCD: central core disease, MmD: multi-minicore disease, CNM: centronuclear myopathy, MHS: malignant hyperthermia susceptibility, CFTD: congenital fiber type disproportion

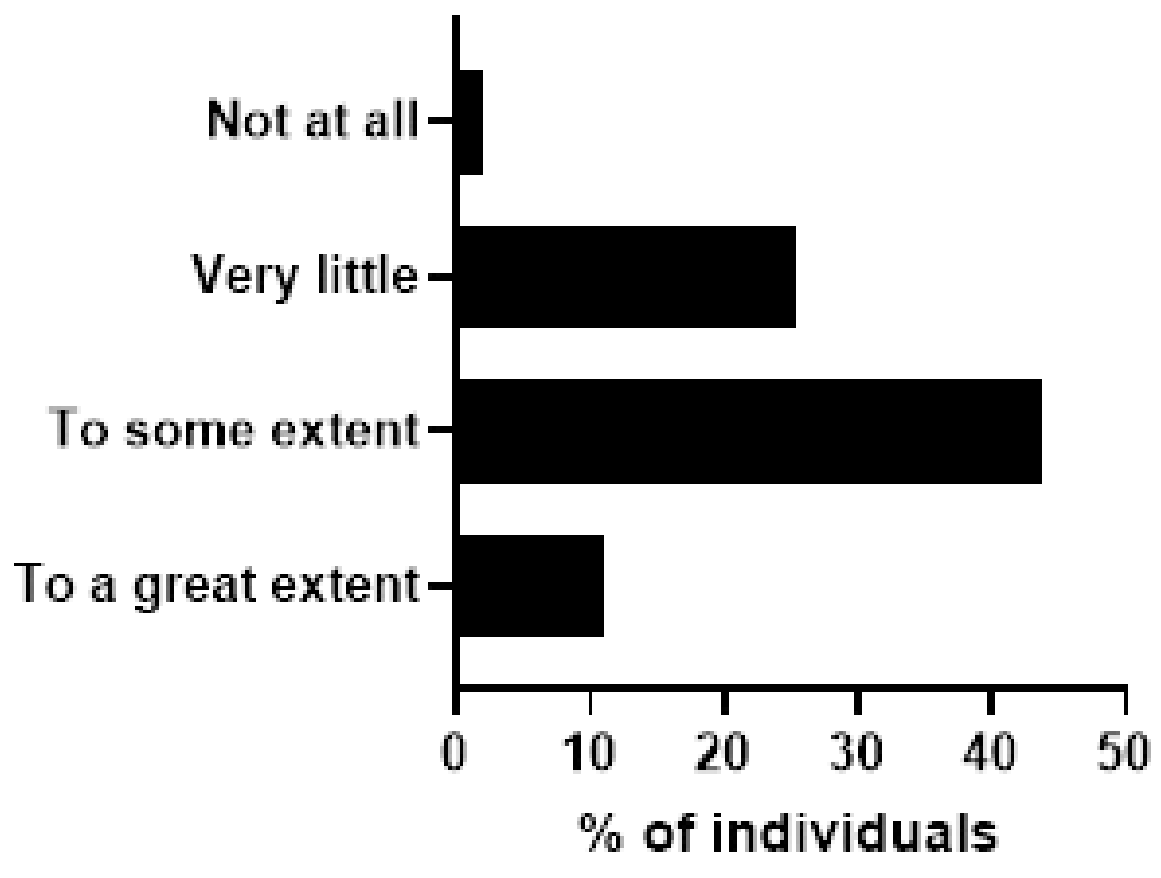

Figure S2: How well do your medications/support measure control your symptoms? The x-axis depicts the percentage of participants that chose that option.

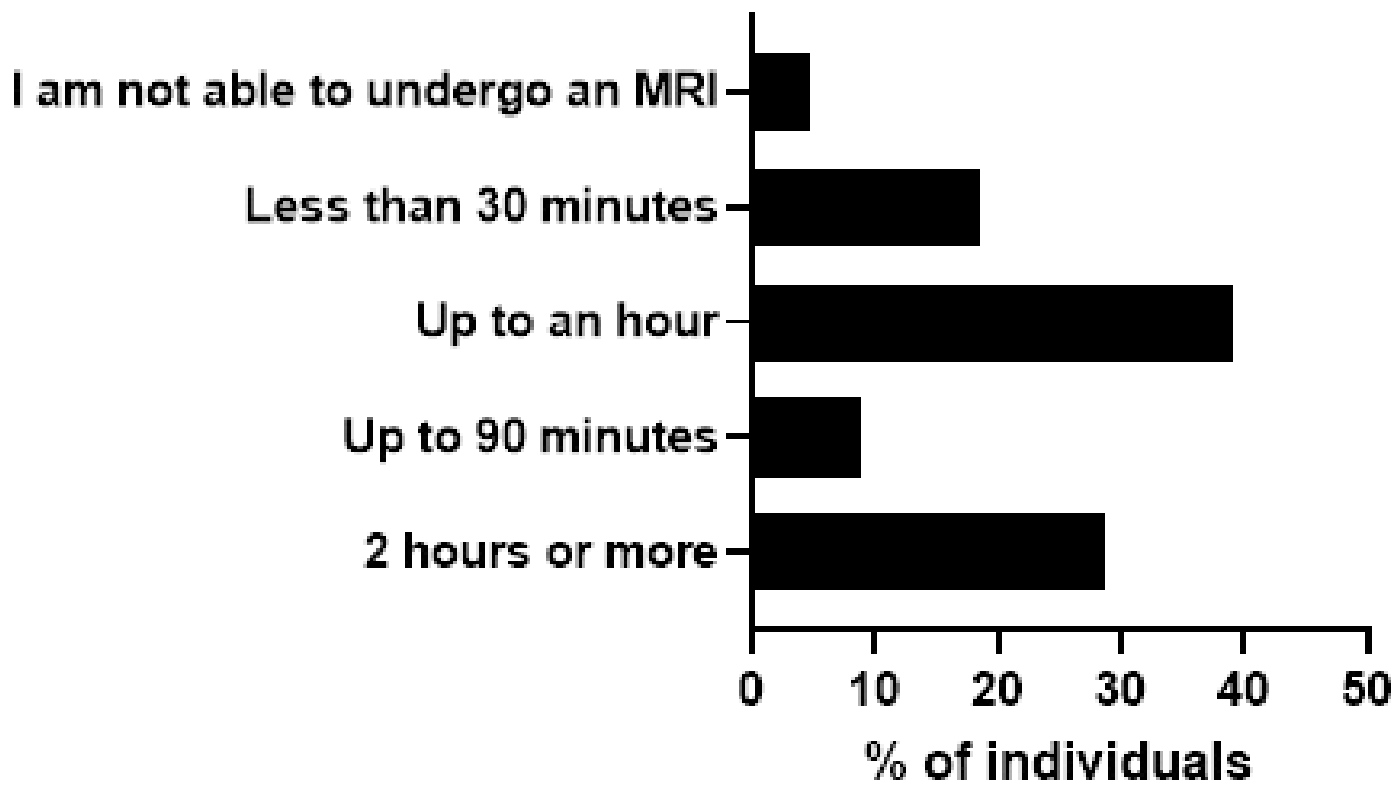

Figure S3: How long would you tolerate lying in an MRI machine? The x-axis depicts the percentage of participants that chose that option.

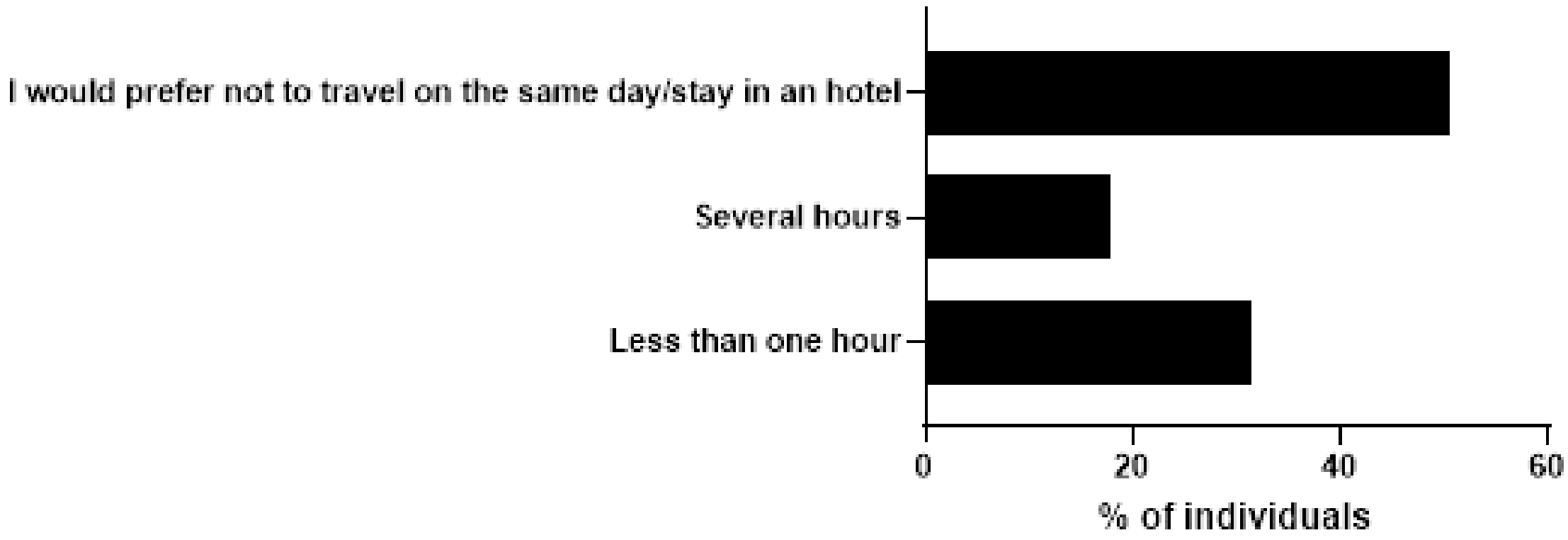

Figure S4: How long would you require to recover from your journey? The x-axis depicts the percentage of participants that chose that option.

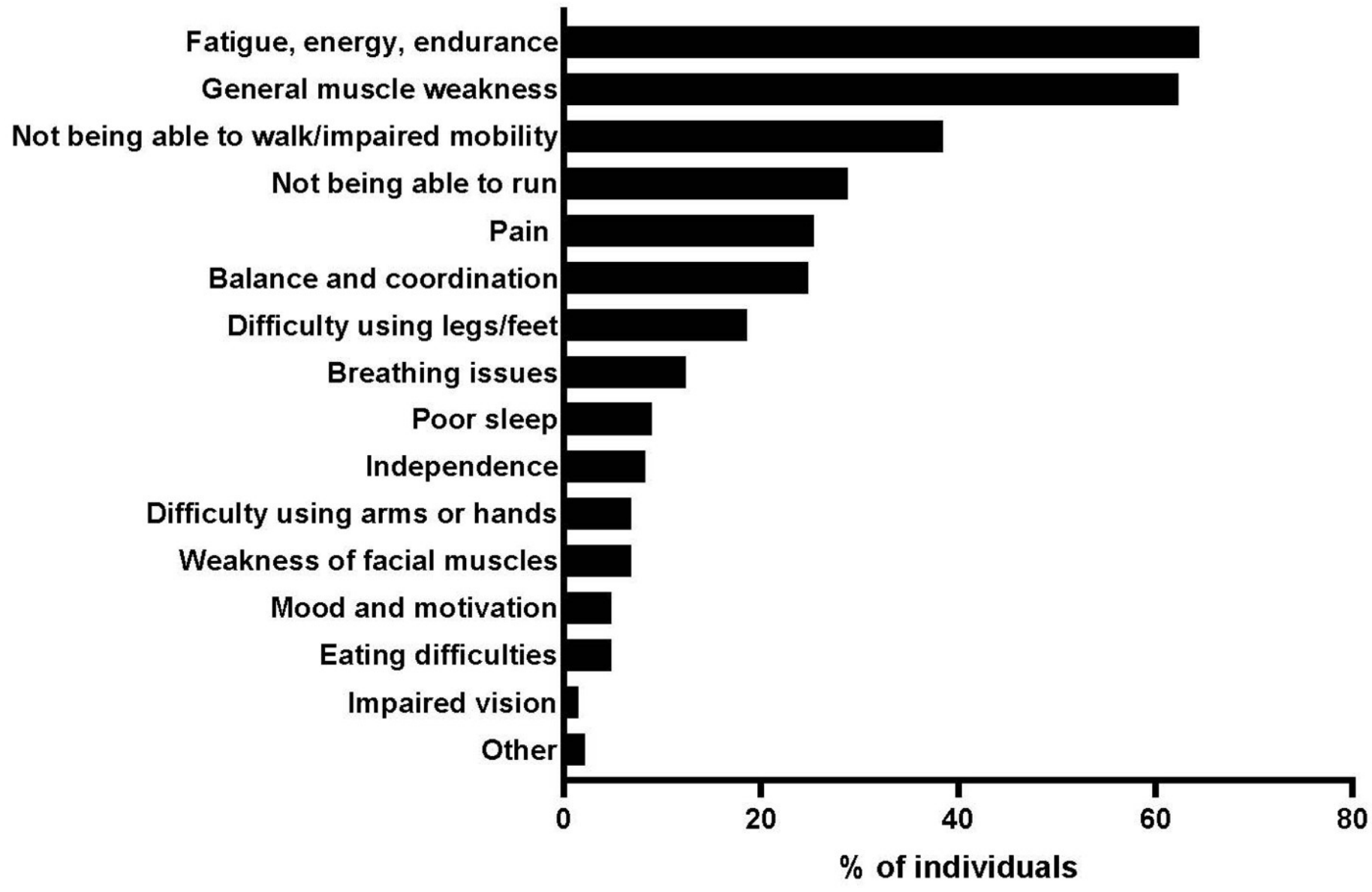

Figure S5: Symptom to be improved first. The x-axis depicts the percentage of participants that chose that symptom.

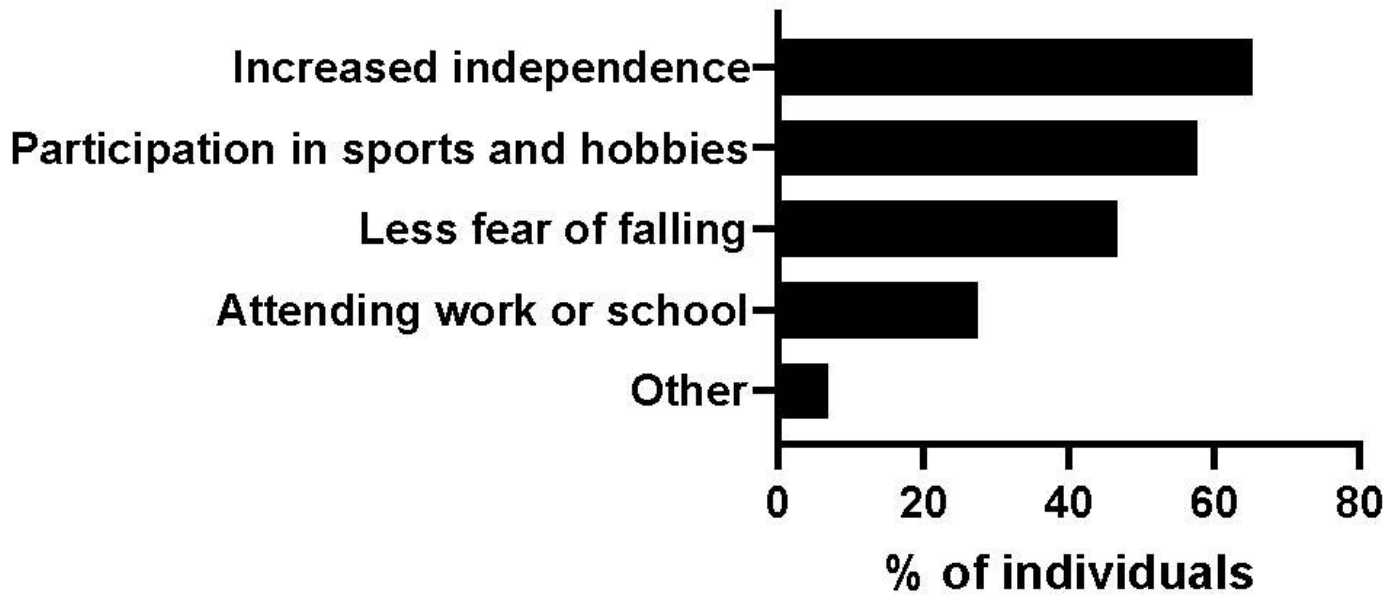

Figure S6: Social benefits of a new therapy. The x-axis depicts the percentage of participants that chose that benefit.

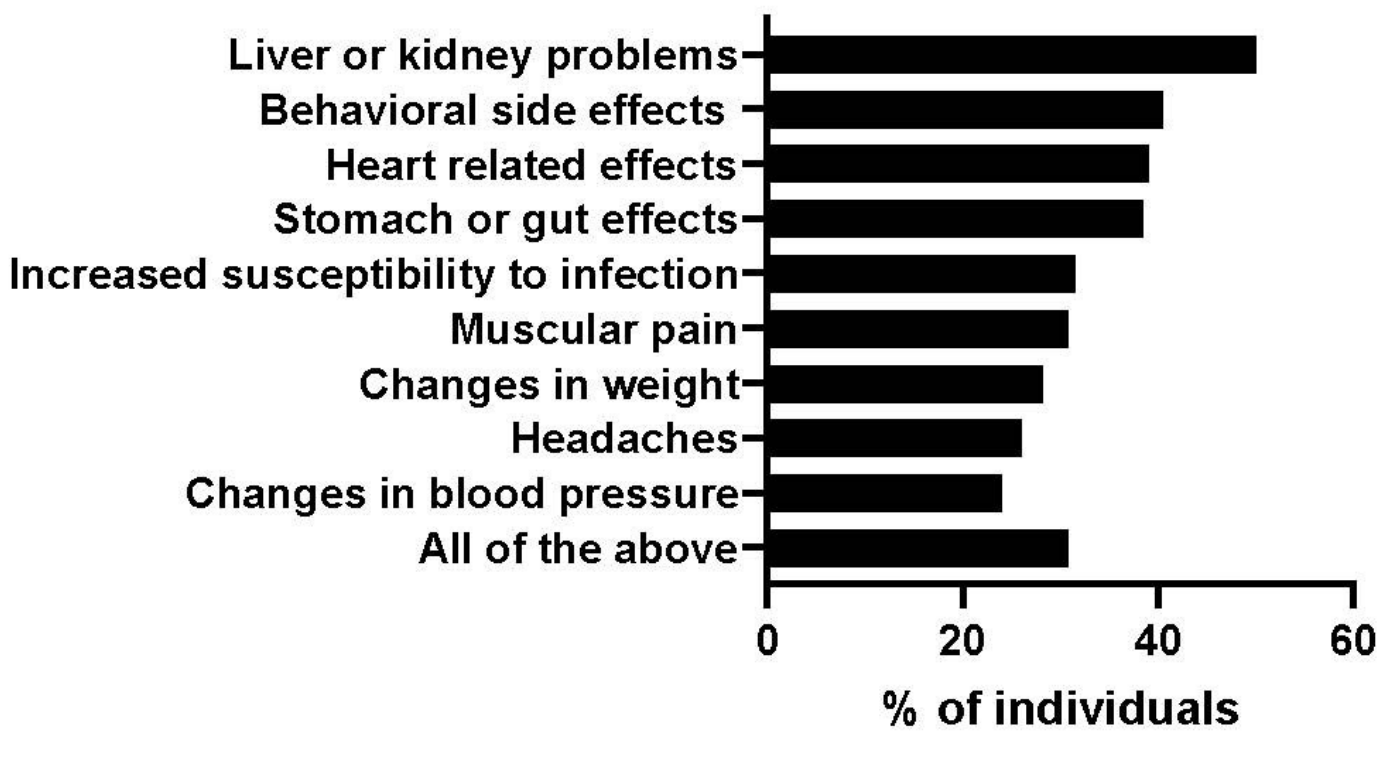

Figure S7: Concerning side effects. The x-axis depicts the percentage of participants that chose that side effect.

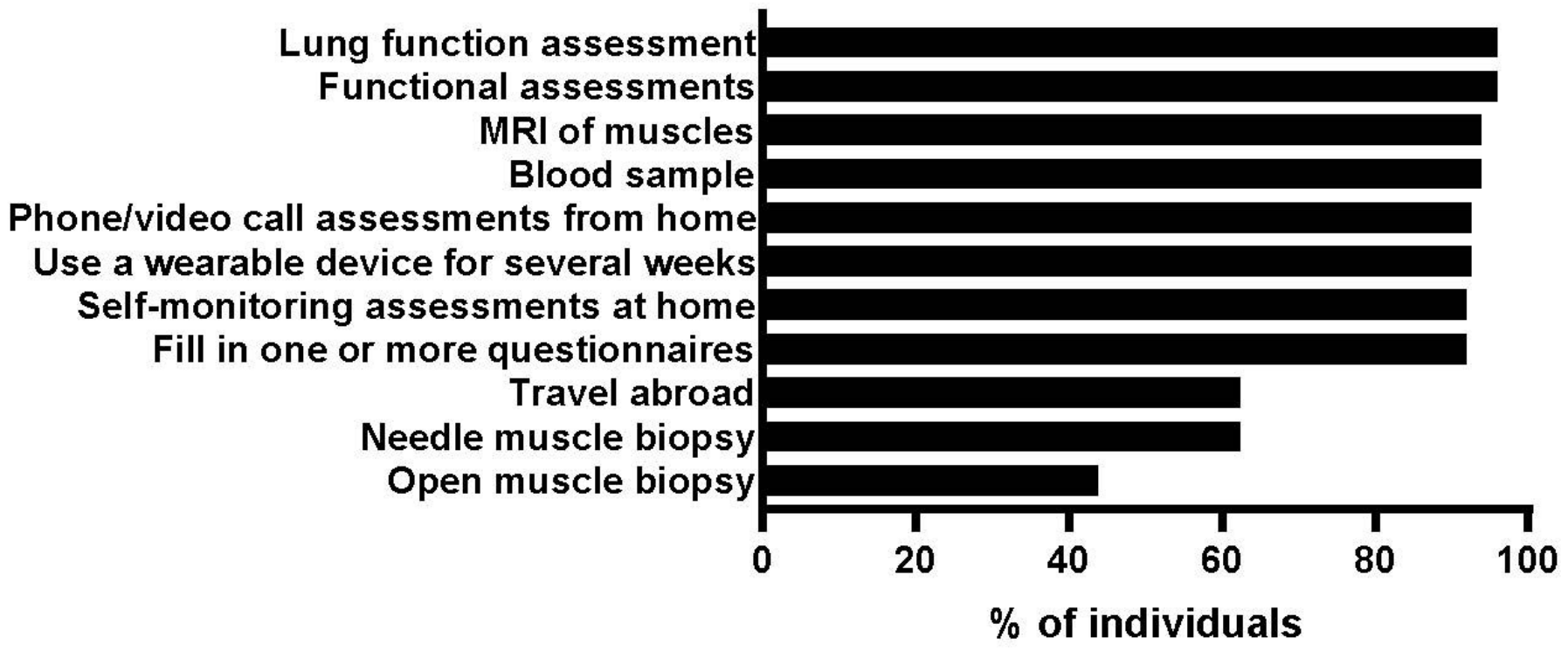

Figure S8: 'What would you be willing to participate in during a clinical trial?' The x-axis depicts the percentage of participants that chose that option.

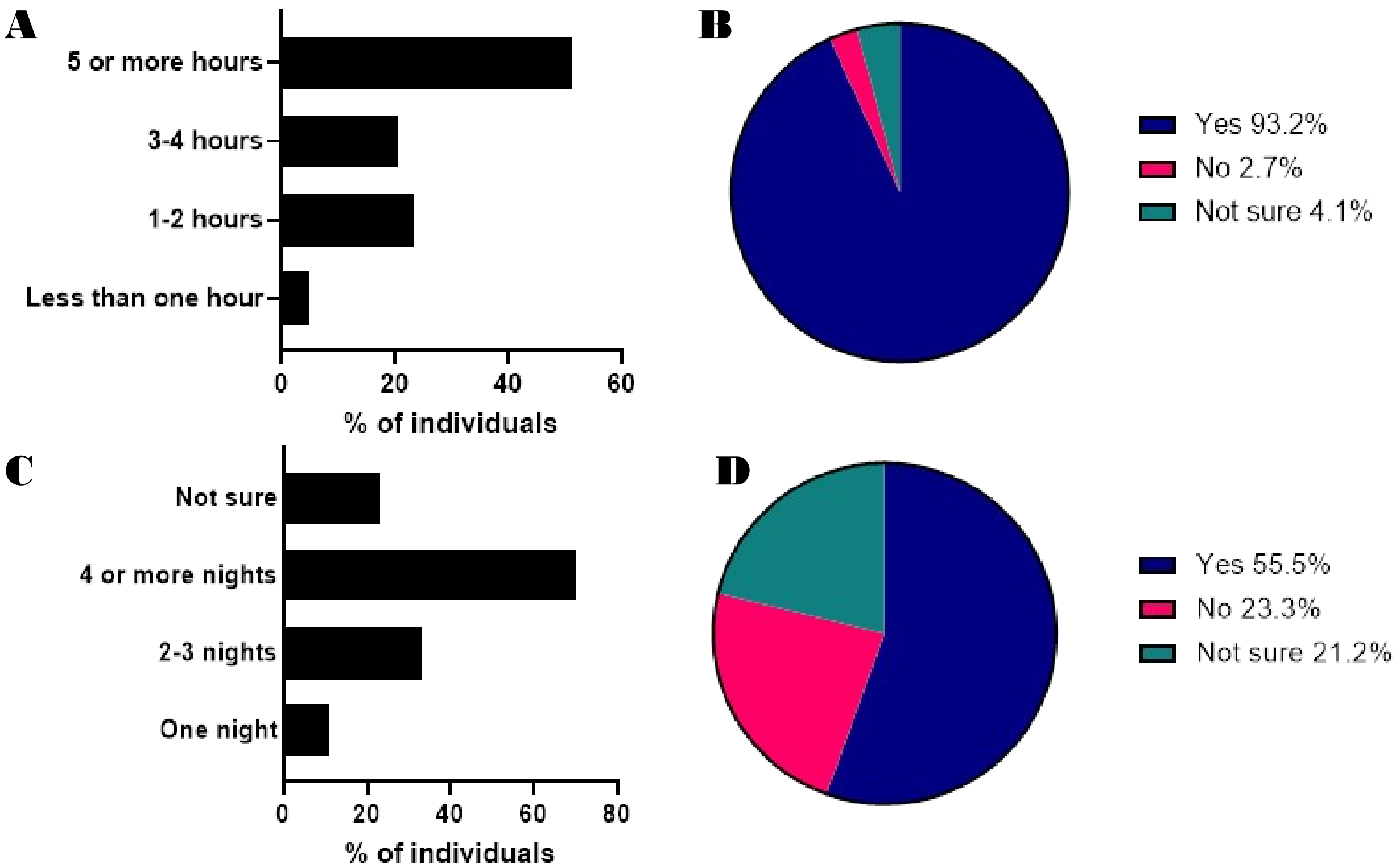

Figure S9: Answers to travel related questions. A: *How long would you be willing to travel? The x-axis depicts the percentage of participants that chose that option. B: Would you be willing to stay overnight? C: How many nights would you be willing to be away from home? The x-axis depicts the percentage of participants that chose that option. D: Would you require a companion to travel with you to a clinical trial?*

Survey S9

RYR-1 Patient Survey

Page 1: Welcome

Welcome to the RYR1-Related Diseases (RYR1-RD) Patient Survey

You have been invited to participate in a research study titled "RYR1 related disease Patient Survey". The purpose of this study is to find out what RYR1 congenital myopathy patients and their caregivers expect from future clinical trials and what would encourage them to participate.

If you agree to take part in this study, you will be asked to complete a survey on the next page. This survey will ask you questions about your condition and how it is currently being managed. It will also ask you your thoughts and expectations on clinical trials and what might help you to decide to participate in the future. The results will be summarized and presented on the website of the RYR-1 Foundation, and at the RYR-1 Foundation international family conference in July 2025. We also aim to publish it in a medical scientific journal so clinicians and researchers can learn from it. This is what we also did with the results of the survey that was performed in 2022. We expect that the results of this survey will enhance the knowledge of patients on future trial participation and of researchers on future trial design.

The survey should take approximately 45 minutes to complete. It is possible to pause in between and finish questions later.

The survey collects limited demographic information. This is treated in an entirely anonymous fashion and the identity of whoever is completing the form cannot be traced.

If you are completing the questionnaire on behalf of another person (a patient), please ensure that you answer all questions—including demographic information—based on the details of the patient, not yourself.

Your participation in this study is completely voluntary and you can withdraw at any time.

Please indicate that you have read and understood this consent form and if you agree to participate in this research study. If the patient is under the age of 18, their parent or legal guardian must complete the consent below on their behalf.

i. I have read and understood this consent form and agree to participate in this study
ii. I no longer wish to participate in this study

Page 2: Participant information

RYR1-RD stands for an RYR1-related disease

1. Why have you chosen to participate in this survey?
   i. I care about the RYR-1-RD community
   ii. I am interested in the study
   iii. I want to help research for RYR-1-RD
   iv. I expect that a trial will start in the near future
   v. Other

1a. If you selected Other, please specify:

2. Where did you hear about this survey?
   i. The RYR-1 Foundation (e.g. newsletter, social media, etc.)
   ii. National patient organization (e.g. newsletter, social media, etc.)
   iii. From your treating physician
   iv. Friend or family member
   v. Other

2a. If you selected Other, please specify:

3. Are you completing this survey as a patient or caregiver?
   i. Patient
   ii. Caregiver
   iii. I am both a patient and caregiver

3a. What is the relationship between you and the patient?

3b. Is the patient also completing this survey?

4. Which country do you currently live in?

5. It is important to capture the ethnicity of the patient to measure the inclusion and diversity within clinical trials. What is the race of the patient?
   i. Arab
   ii. Asian
   iii. African
   iv. Mixed or multiple ethnic groups
   v. Caucasian
   vi. Hispanic/Latino/Caribbean
   vii. Prefer not to say
   viii. Other

5a. If you selected Other, please specify:

6. Current age (of the patient):

7. (Biological) sex of the patient
   i. Female
   ii. Male
   iii. Prefer not to say

8. Highest educational level (so far):
   i. Preschool

ii. Primary school
iii. Secondary school
iv. Vocational education and training
v. College/university (Bachelor)
vi. College/university (Master)
vii. PhD/MD/JD (doctoral level)

9. Employment status
   i. (Volunteer)work fulltime (>32h/week)
   ii. (Volunteer)work parttime (<32h/week)
   iii. Retired
   iv. Unable to work
   v. Still in school

10. Social status
   i. Living alone
   ii. Living with partner/family members
   iii. Living with other housemates
   iv. Assisted living

11. What type of RYR1-RD do you have?
   i. Centronuclear Myopathy (CNM)
   ii. Central Core Disease (CCD)
   iii. Multiminicore Disease (MmD)
   iv. Congenital fiber-type disproportion (CFTD)
   v. Nemaline Myopathy (NM)
   vi. Malignant hyperthermia susceptibility (MHS)
   vii. Other
   viii. Unknown

11a. If you selected Other, please specify:

12. What is the inheritance of your RYR1-RD?
   i. Autosomal dominant
   ii. Autosomal recessive
   iii. De novo/spontaneous
   iv. Unknown

13. Do you have any other family members with RYR1-RD?
   i. Yes
   ii. No

13a. If Yes, specify:

Page 3: Current management of RYR-1-RD

14. Are you taking any medication or supplements for RYR-1-RD specific issues or related symptoms?
   i. No, never
   ii. Yes, in the past

iii. Yes, currently

15. What medications are you currently taking to help manage your RYR-1-RD condition, if any? Select all that apply.
    i. Nothing
    ii. Salbutamol and/or pyridostigmine
    iii. Anti-anxiety medication
    iv. Pain killers
    v. Other prescription medication
    vi. Over the counter medication such as paracetamol, ibuprofen or tylenol
    vii. Dietary and herbal supplements
    viii. Other

15a. If you selected other, please specify:

16. Other than medication, are you using any of the following support measures to help manage your RYR-1-RD condition? Select all that apply.
    i. Not currently using anything
    ii. Exercise
    iii. Physical or occupational therapy
    iv. Braces, splints, kinesio tape, etc.
    v. Mobility aids (such as a walker or wheelchair)
    vi. Electric bike
    vii. (Nocturnal) mechanical ventilation
    viii. Surgery (such as scoliosis surgery)
    ix. Diet modification
    x. Counselling/therapy
    xi. Adjustments to the house
    xii. Adjustments to the car
    xiii. Other

16a. If you selected Other, please specify:

17. How well do you think that your medications and support measures control your overall condition and symptoms?
    i. I am not using any treatments or other therapies
    ii. Not at all
    iii. Very little
    iv. To some extent
    v. To a great extent

18. What are the biggest limitations of your medications and support measures? Select all that apply.
    i. N/A I am not using any treatments
    ii. Not very effective
    iii. High cost
    iv. Limited availability or accessibility (e.g., do not live close to a specialist center)
    v. Number of medications that I have to take daily
    vi. Time consuming
    vii. Requires a lot of effort
    viii. Other

18a. If you selected Other, please specify:

Page 4: Information about clinical trials

In the next section of the survey, we are going to ask about your thoughts and expectations of clinical trials.

Clinical trials are research studies involving people to test whether particular treatments are safe and how well they work.

If you take part in a clinical trial, you may be one of the first people to receive a new treatment. There's a chance that the new treatment turns out to be no better, or worse, than the standard treatment. It's also possible you'll experience unexpected side effects.

You could also be given a placebo. A placebo is used in clinical trials to test the effectiveness of treatments and is most often used in drug studies. For instance, people in one group get the tested drug, while the others receive a fake drug, or placebo, that they think is the real thing. Alternatively, there may be two portions of the trial where one includes the active drug and one the placebo, and you will receive one or the other first, then switch in a blinded fashion. This way, the researchers can measure if the drug works by comparing how both groups react. If they both have the same reaction — improvement or not — the drug is deemed not to work. Placebo-controlled trials will be required by the regulatory authorities for approval.

You may have to visit the place where the treatment is given more often, or have more tests, including measuring blood levels of the drug, treatments or monitoring. You may also need to travel to a specialist center to take part in the clinical trial.

It's important to find out about the inconvenience and risks involved before you decide to participate in a clinical trial, and to carefully weigh up whether it's worth it.

Bear in mind:

- It can be time consuming – you may be expected to attend a number of screening and follow-up sessions, and some trials require you to stay overnight
- Some of the tests can be tiring, or take a long time to complete
- Some of the tests could be painful, such as a muscle biopsy
- There may be restrictions on what you can and cannot do – for example, you may be asked to not eat, or not drink alcohol, for a period of time
- You may experience unknown side effects from the treatment

For further information you can visit the following links

- NHS – Clinical trials
- National Institutes of Health – What are clinical trials?

Page 5: Thoughts on clinical trials

19. Have you ever participated in a clinical trial/study?
    i. Yes
    ii. No

19a. How did you experience this?

   i. Positive

ii. Neutral
iii. Negative

19b. Please elaborate on your answer:

20. Would you consider participating in a clinical trial/study?
   i. Yes
   ii. No
   iii. Not sure

Page 6: Encouraging factors in clinical trials

21. What would encourage you to participate in a clinical trial? Please rank the following from 0 (not encouraging) to 5 (most encouraging).
Please don't select more than 1 answer(s) per row.

	0 (Not encouraging)	1	2	3	4	5 (Most encouraging)
Compensation for travel/time						
Access to new investigational product or therapy						
Help in organizing my visit (e.g. booking hotels and transport for me)						
Access to trial results when published						
To be seen by a specialist center						
Flexibility in appointment times						
Meet other patients with the same disease						
Benefits for the RYR-1-RD Community						
Benefits for my family						
Clear indication of level of commitment required						

21a. Is there anything else that might encourage you to participate in a clinical trial that was not listed here?

Page 7: Discouraging factors in clinical trials

22. What would discourage you from participating in a clinical trial? Please rank the following from 0 (not discouraging) to 5 (most discouraging).
Please don't select more than 1 answer(s) per row.

	0 (Not discouraging)	1	2	3	4	5 (Most discouraging)
Fear of side effects from treatment						
Lack of transportation to get to trial site						
I could be in the placebo group						
Too many appointments are required						
Facility of the clinical trial is far away						
Lack of flexibility in appointment times						
Would have to miss work/school						
Lack of financial compensation for time spent on the trial						
Do not want RYR-1-RD diagnosis on medical records						
Travel is expensive						
Long time to wait between trial and results						

22a. Is there anything else that might discourage you from participating in a clinical trial that was not listed here?

Page 8: Practical aspects of trial participation

23. What would you be willing to participate in during a clinical trial? Select all that apply.
    i. Blood sample
    ii. Open muscle biopsy (An open biopsy involves a small incision through the skin and into the muscle, so that a sample of muscle tissue can be removed from the affected area)
    iii. Needle muscle biopsy (A needle biopsy involves inserting a needle into the muscle. When the needle is removed, a small piece of tissue remains in the needle. More than one needle stick may be needed to get a large enough sample.)
    iv. MRI of muscles
    v. Functional assessments e.g. grip strength, timed up and go, stair climb
    vi. Lung function assessment (A test used to check how well your lungs work. It measures how much air you breathe in, how much you breathe out and how quickly you breathe out)
    vii. Use a wearable device such as a smartwatch for several weeks
    viii. Fill in one or more questionnaires
    ix. Other

23a. If you selected Other, please specify:

23b. During a clinical trial, it can be important to monitor the effects that a new therapy might have. You could therefore be subjected to a test multiple times. How many needle muscle biopsies would you be willing to have?
    i. One muscle biopsy only
    ii. Two or more muscle biopsies

23c. How long would you tolerate lying in an MRI machine?
    i. Less than 30 minutes
    ii. Up to an hour
    iii. Up to 90 minutes
    iv. 2 hours or more

24. Is there a procedure that would discourage you from taking part in a clinical trial? Select all that apply.
    i. Blood sample
    ii. Needle muscle biopsy
    iii. Open muscle biopsy
    iv. MRI machine
    v. Functional assessments e.g. grip strength, timed up and go
    vi. Lung function assessments
    vii. Use a wearable device e.g. fitbit for several weeks
    viii. Fill in one or more questionnaires
    ix. Other

24a. If you selected Other, please specify:

25. Some of the tests during a clinical trial could be strenuous and take a lot of effort. If you had traveled a long way to take part in a clinical trial, how long would you require to recover from your journey before taking part in these tests?
    i. Less than one hour
    ii. Several hours

iii. I would prefer not to travel on the same day as the tests (Arrive the day before and stay at a hotel)

26. How long would you be willing to travel to take part in a clinical trial?
    i. Less than one hour
    ii. 1-2 hours
    iii. 3-4 hours
    iv. 5 or more hours

27. Would you be willing to stay overnight in a hotel that was suitably equipped to meet your needs?
    i. Yes
    ii. No
    iii. Not sure

27a. How many nights would you be willing to be away from home?
    i. One night
    ii. 2-3 nights
    iii. 4 or more nights
    iv. Not sure

28. Would you be willing to travel abroad for a clinical trial?
    i. Yes
    ii. No
    iii. Not sure

28a. Would you be willing to travel to a country with a different language?
    i. Yes
    ii. No
    iii. Not sure

29. Would you require a companion to travel with you to a clinical trial? (e.g., parent, partner or friend)
    i. Yes
    ii. No
    iii. Not sure

30. Would you be willing to complete self-monitoring assessments at home as a part of a clinical trial? E.g., Complete a strength test and send in the results electronically
    i. Yes
    ii. No
    iii. Not sure

31. Would you be willing to take part in phone/video call assessments from home?
    i. Yes
    ii. No
    iii. Not sure

32. Would your relatives/friends be willing to participate in a clinical trial which included people with and without RYR-1-RD?
    i. Yes
    ii. No

iii. Not sure

Page 9: Expectations of clinical trials

33. Is there something that you have lost the ability to do because of your RYR-1-RD, that you would like to regain?

34. Is there a particular symptom of RYR-1-RD that you would like to be improved first? Select up to 3 options. (PS in question 51 we will ask you about your current symptoms and provide the same list of options)
    i. Pain
    ii. Fatigue, Energy and Endurance
    iii. Not being able to walk or impaired mobility
    iv. Not being able to run
    v. Difficulty using arms or hands
    vi. Difficulty using legs/feet
    vii. General muscle weakness
    viii. Weakness of facial muscles
    ix. Balance and coordination
    x. Mood and motivation (e.g., depression or anxiety)
    xi. Breathing issues
    xii. Poor sleep
    xiii. Impaired vision
    xiv. Independence
    xv. Eating difficulties
    xvi. Other

34a. If you selected Other, please specify:

35. What do you feel is the most important outcome of a new therapy? Select up to 3 options.
    i. Stopping the progression of the disease
    ii. Reduction in pain
    iii. Reduction in fatigue, with increased energy and endurance
    iv. Regaining strength
    v. Preserving or improving respiratory and lung function
    vi. Strengthening facial muscles
    vii. Improved balance and coordination
    viii. Improved mobility
    ix. Improved mood and motivation
    x. Reducing fall risk
    xi. Increased independence
    xii. Other

35a. If you selected Other, please specify:

36. What is your minimum expectation for a clinical trial?
    i. Improving research of RYR-1-RD
    ii. Feedback of the results
    iii. Being seen in a specialist center
    iv. Other

36a. If you selected Other, please specify:

37. What social benefits of a therapy would you consider most important? Select up to 3 options.
    i. Increased independence
    ii. Less fear of falling
    iii. Attending work or school
    iv. Participation in sports and hobbies
    v. Other

37a. If you selected Other, please specify:

38. If a treatment was available that could potentially help your symptoms of RYR-1-RD, which side effect would you be concerned about experiencing? Select all that apply.
    i. Headaches
    ii. Muscular pain
    iii. Stomach or gut effects, such as nausea, lack of appetite or diarrhea
    iv. Liver or kidney problems
    v. Increased susceptibility to infection
    vi. Changes in blood pressure
    vii. Behavioral side effects such as change in mood; anxiety, insomnia etc.
    viii. Heart related effects such as palpitations
    ix. Changes in weight
    x. All of the above
    xi. Other

38a. If you selected Other, please specify:

39. How would you prefer to receive updates or results of a clinical trial?
    i. Newsletter
    ii. Website
    iii. Post
    iv. E-mail
    v. Phone call

40. Do you have any other thoughts, expectations, or concerns about taking part in a clinical trial that you haven't had an opportunity to share in this survey?

41. How do you receive information about RYR-1-RD related topics and updates on clinical trials?
    i. The RYR-1 Foundation (e.g. newsletter, social media, etc.)
    ii. National patient organization (e.g. newsletter, social media, etc.)
    iii. Rarediseases.org (NORD)Updates from your treating physician
    iv. Other
    v. I do not receive any information

41a. If you selected Other, please specify:

41b. Do you find this information sufficient?
    i. Yes
    ii. No

41c. If you selected No, what information do you miss or what could be improved?

Page 10: Information about current condition

42. At what age did symptoms first appear?

43. What was the first symptom experienced?
    i. Weakness in arms/hands
    ii. Pain
    iii. Fatigue
    iv. Weakness in legs/feet
    v. Other

43a. If you selected Other, please specify:

44. RYR-1-RD may be diagnosed based upon a clinical examination, identifying key characteristics of the condition, family history and genetic testing. Have you received a diagnosis of RYR-1-RD from a healthcare professional?
    i. Yes
    ii. No

44a. At what age was the diagnosis given?

44b. Has the diagnosis been genetically confirmed?

    i. Yes
    ii. No

45. How would you describe the current severity of your disease overall?
    i. Not at all affected
    ii. Mildly affected
    iii. Moderately affected
    iv. Severely affected

46. How do you feel that your condition has changed in the last 6 months?
    i. Very much improved
    ii. Much improved
    iii. Minimally improved
    iv. No change
    v. Minimally worse
    vi. Much worse
    vii. Very much worse

47. How do you feel that your condition has changed in the last 3 years?
    i. Very much improved
    ii. Much improved
    iii. Minimally improved
    iv. No change
    v. Minimally worse
    vi. Much worse
    vii. Very much worse

48. Which of the following are you able to do? Select all that apply
    i. Hold head up without support

ii. When lying down, I can roll onto my side without assistance
iii. I can balance myself by sitting up straight without using my arms or hands for at least 10 seconds
iv. Crawl on hands and knees for at least three movements (e.g., move arms and legs three times)
v. I can stand up for at least 10 seconds without support
vi. I can stand up for at least 10 seconds, but I would need support (e.g., holding onto a chair)
vii. I can walk at least 100 meters/109 yards without assistance
viii. I can walk at least 10 meters/ 11 yards without assistance
ix. I can walk at least 5 steps without assistance
x. I can walk at least 5 steps, but I would need to hold on to something for support
xi. I can climb at least 4 stairs independently (holding on to a railing)
xii. I can use my hands to hold pencil, drive wheelchair or use phone keypad
xiii. When sitting, I can raise both arms at the same time above my head
xiv. When sitting, I can raise one or both of my hands to my mouth

Page 11: Difficulties with activities

49. Do you have any difficulty with the activities listed below because of problems with your arms or hands? Please rank from 0 (extreme difficulty or unable to perform activity) to 4 (no difficulty).
Please don't select more than 1 answer(s) per row.

	N/A	0 (extreme difficulty or unable to perform activity)	1	2	3	4 (no difficulty)
Any of your work, housework, or school activities						
Your hobbies						
Your recreational or sporting activities						
Lifting a bag of groceries to your waist level						
Lifting a bag of groceries above your head						
Grooming your hair						
Pushing up on your hands (e.g. from bathtub or chair)						
Preparing food (e.g. peeling, cutting)						
Driving						
Vacuuming, sweeping, or raking						
Dressing						
Doing up buttons						
Opening doors						
Cleaning						
Tying laces on shoes						
Sleeping						
Laundering clothes (e.g. washing, ironing, folding)						
Opening a jar						
Throwing a ball						
Carrying a small suitcase						

Page 12: Functional abilities

50. How is your walking ability?

i. Not at all affected
ii. I can walk independently with aids, such as a cane, a walker or orthosis and do not use a wheelchair
iii. I can walk independently with aids, such as a cane, a walker or orthosis and I use a wheelchair outdoors.
iv. I use the wheelchair indoors and outdoors, but can walk short distances (with or without aids)
v. I am unable to walk: wheelchair dependent

50a. What walking aids do you use, if any? Select all that apply.

i. Walking stick
ii. Splints
iii. Walking frame
iv. Manual wheelchair
v. Powered wheelchair/scooter

51. How do you feel your respiratory (breathing) health has been affected by RYR-1-RD?

i. Not affected
ii. Mildly affected
iii. Moderately affected - require non-invasive ventilation (e.g., BiPAP machine)
iv. Severely affected - require invasive ventilation

51a. If you require ventilation, is this full time or part time?

i. N/A I do not require ventilation
ii. Part time - less than 16 hours per 24 hours
iii. Full time - 16 hours or more per 24 hours

<u>Page 13: Symptoms that cause you concern</u>

52. Which symptoms of your condition cause you concern?
    Please rank from 0 (no concern) to 5 (very concerned).

Please don't select more than 1 answer(s) per row.

	0 (No concern)	1	2	3	4	5 (Very concerned)
Pain						
Fatigue, energy and endurance						
Not being able to walk or impaired mobility						
Not being able to run						
Difficulty using arms or hands						
General muscle weakness						
Facial weakness						
Balance and coordination						
Mood and motivation (e.g., depression or anxiety)						
Breathing issues						
Poor sleep						
Impaired vision						
Eating difficulties						

52a. Are there symptoms not mentioned in this list which cause concern?

Page 14: Symptoms that cause difficulty in daily activities

53. Which symptoms of your RYR-1-RD causes difficulty in daily activities? Please rank from 0 (no difficulty) to 5 (causes great difficulty)
Please don't select more than 1 answer(s) per row.

	0 (No difficulty)	1	2	3	4	5 (Causes great difficulty)
Pain						
Fatigue, energy and endurance						
Not being able to walk or impaired mobility						
Not being able to run						
Difficulty using arms or hands						
General muscle weakness						
Facial weakness						
Balance and coordination						
Mood and motivation (e.g., depression or anxiety)						
Breathing issues						
Poor sleep						
Impaired vision						
Eating difficulties						

53a. Are there any other symptoms that hinder you greatly that are not mentioned in this list?

54. Could you describe in more detail how these symptoms impact your daily life?

Page 15: Concerns for future

55. Thinking about the future, what concerns you most about your condition? Please rank from 0 (not at all concerned) to 5 (causes great concern).
Please don't select more than 1 answer(s) per row.

	0 (Not at all concerned)	1	2	3	4	5 (Causes great concern)
The stress of not knowing how my disease will progress						
Losing independence						
Losing mobility/ability to walk						
Developing respiratory issues						
Not having the energy to work or live as I want to						
Coping with chronic pain						
Financial concerns						
Becoming a burden to my family						
Losing social connections						
Fear of seeing family affected						

55a. Is there anything not mentioned in this list that you would like to add?

55b. Could you describe this concern in more detail?

Page 16: Final questions

56. Do you have any diagnosed illnesses or conditions, alongside your RYR-1-RD?

57. Is there any aspect of your condition or treatment that you feel is not covered in this survey?

58. What topics would you like future research to focus on?

If you want to receive information about this study once it is completed, please send an email to the researcher.
